# *Tamarindus indica* Extract Alters Release of Alpha Enolase, Apolipoprotein A-I, Transthyretin and Rab GDP Dissociation Inhibitor Beta from HepG2 Cells

**DOI:** 10.1371/journal.pone.0039476

**Published:** 2012-06-19

**Authors:** Ursula Rho Wan Chong, Puteri Shafinaz Abdul-Rahman, Azlina Abdul-Aziz, Onn Haji Hashim, Sarni Mat Junit

**Affiliations:** 1 Department of Molecular Medicine, Faculty of Medicine, University of Malaya, Kuala Lumpur, Malaysia; 2 University of Malaya Centre for Proteomics Research, Medical Biotechnology Laboratory, Faculty of Medicine, University of Malaya, Kuala Lumpur, Malaysia; Aligarh Muslim University, India

## Abstract

**Background:**

The plasma cholesterol and triacylglycerol lowering effects of *Tamarindus indica* extract have been previously described. We have also shown that the methanol extract of *T. indica* fruit pulp altered the expression of lipid-associated genes including *ABCG5 and APOAI* in HepG2 cells. In the present study, effects of the same extract on the release of proteins from the cells were investigated using the proteomics approach.

**Methodology/Principal Findings:**

When culture media of HepG2 cells grown in the absence and presence of the methanol extract of *T. indica* fruit pulp were subjected to 2-dimensional gel electrophoresis, the expression of seven proteins was found to be significantly different (*p*<0.03125). Five of the spots were subsequently identified as alpha enolase (ENO1), transthyretin (TTR), apolipoprotein A-I (ApoA-I; two isoforms), and rab GDP dissociation inhibitor beta (GDI-2). A functional network of lipid metabolism, molecular transport and small molecule biochemistry that interconnects the three latter proteins with the interactomes was identified using the Ingenuity Pathways Analysis software.

**Conclusion/Significance:**

The methanol extract of *T. indica* fruit pulp altered the release of ENO1, ApoA-I, TTR and GDI-2 from HepG2 cells. Our results provide support on the effect of *T. indica* extract on cellular lipid metabolism, particularly that of cholesterol.

## Introduction

*Tamarindus indica*, also known as tamarind, is a tropical fruit tree that grows naturally in many tropical and subtropical regions. Due to the sour taste, its fruit pulp is widely used to add flavour in cooking. Many claims have been made on the medicinal use of tamarind fruit pulps including as gentle laxative, expectorant, anti-pyretic and antimicrobial agents [1], [2], [3]. Biochemical experiments have also shown that tamarind extracts possess high antioxidant activities [4], [5]. In addition, the fruit pulp extract of *T. indica* has also been shown to cause a decrease in the levels of serum total cholesterol and triacylglycerol but an increase in the HDL cholesterol levels in hypercholesterolaemic hamsters [4] and in humans [5]. However, the precise mechanisms of action at the molecular levels have yet to be deciphered.

Analysis of the methanol extract of the tamarind fruit pulp by HPLC revealed the predominant presence of proanthocyanidins, including (+)-catechin and (–)-epicatechin [6]. The jasmine green tea epicatechin has been shown to reduce the levels of triacylglycerol and cholesterol in the sera of hamsters fed with a high-fat diet [7]. The observed hypolipidaemic effects of epicatechin were postulated to involve inhibition of the absorption of dietary fat and/or cholesterol or through the reabsorption of bile acids since it did not inhibit liver HMGCoA reductase [7]. More recently, we have shown that the methanol extract of *T. indica* fruit pulps significantly up-regulated the expression of a total of 590 genes and down-regulated 656 genes expression in HepG2 cells [8]. Amongst the genes that were altered in expression were those that encode proteins associated with lipoprotein metabolism, including ApoA-I, ApoA-IV, ApoA-V and ABCG5 but not the HMGCoA reductase. Both ApoA-I and ABCG5 are involved in the reverse cholesterol transport, where the latter, together with ABCG8, are involved in the hepatobiliary cholesterol secretion.

In the present study, we have investigated the effects of *T. indica* fruit pulp extract on the release of proteins from HepG2 cells as a mean to validate previously reported gene expression data at the protein level. Identification of proteins that were differently altered in the cell culture media may help to improve our understanding of the metabolic pathways that are affected and the molecular mechanisms involved. Secreted proteins were specifically targeted in this study as they may be involved in regulating the many biological processes throughout the human body, including lipid metabolism.

**Figure 1:**
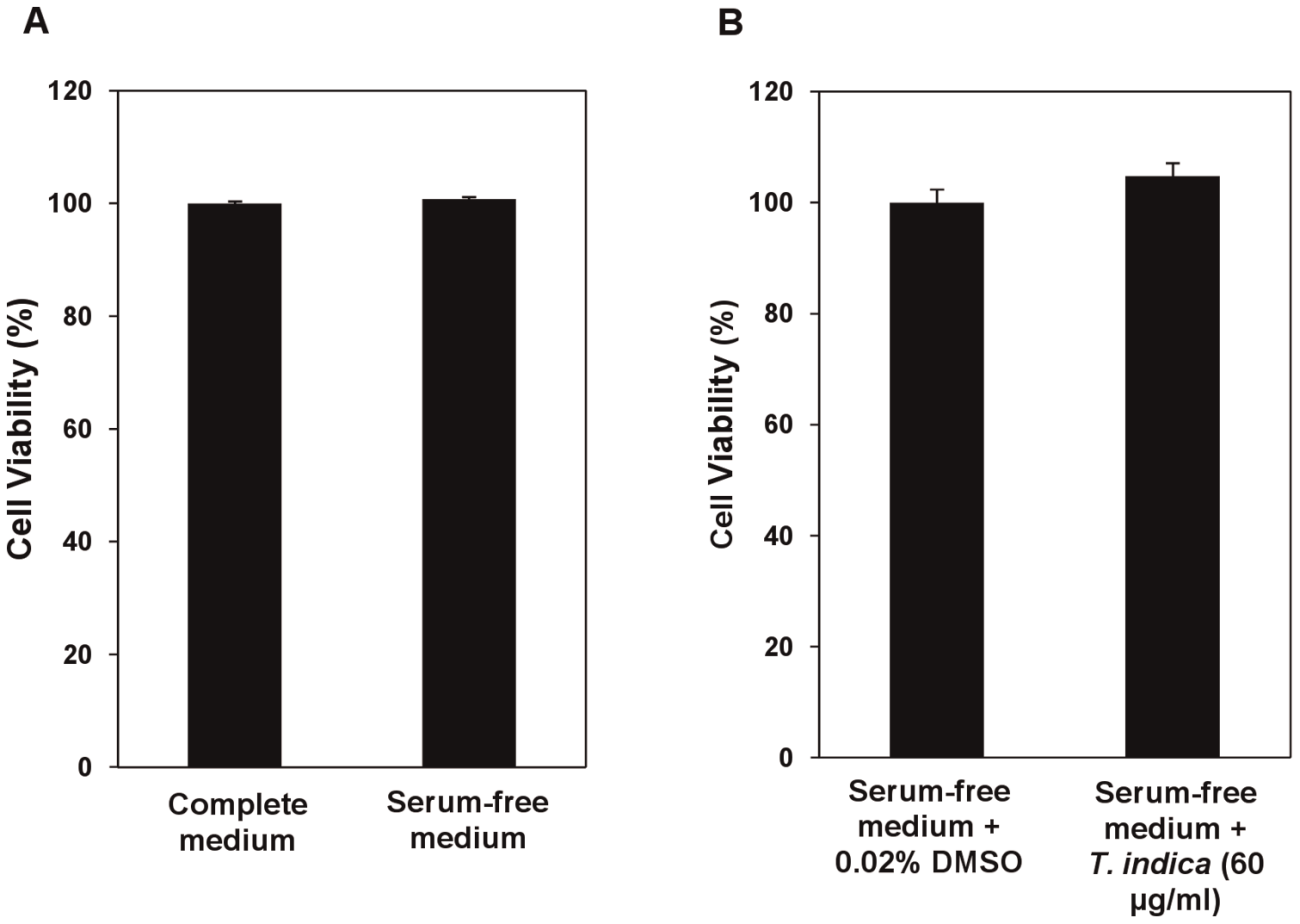
MTT analysis to assess cell viability. HepG2 cells were grown in complete or serum-free media (A) and in serum-free media in the presence of vehicle, 0.02% DMSO (control) or 60 µg/ml methanol extract of the *T. indica* fruit pulp (B). Assays were done in triplicate and all data are expressed as mean ± S.E.M.

## Materials and Methods

### Preparation of methanol extract of the *T. indica* fruit pulp

The *T. indica* fruit pulp extract was prepared as previously described [8], with slight modifications. Briefly, ripe fruit pulp of *T. indica* was separated from the seeds, air-dried and then powdered. The powdered fruit pulp (10 g) was then placed in a conical flask and soaked in 200 ml methanol at room temperature (RT). The mixture was then stirred with a magnetic stirrer for 1 h and kept in the dark for 24 h The resulting extract was then filtered and dried in a rotary evaporator and finally redissolved in 10% DMSO. Samples were kept at −20°C until further analysis.

**Figure 2:**
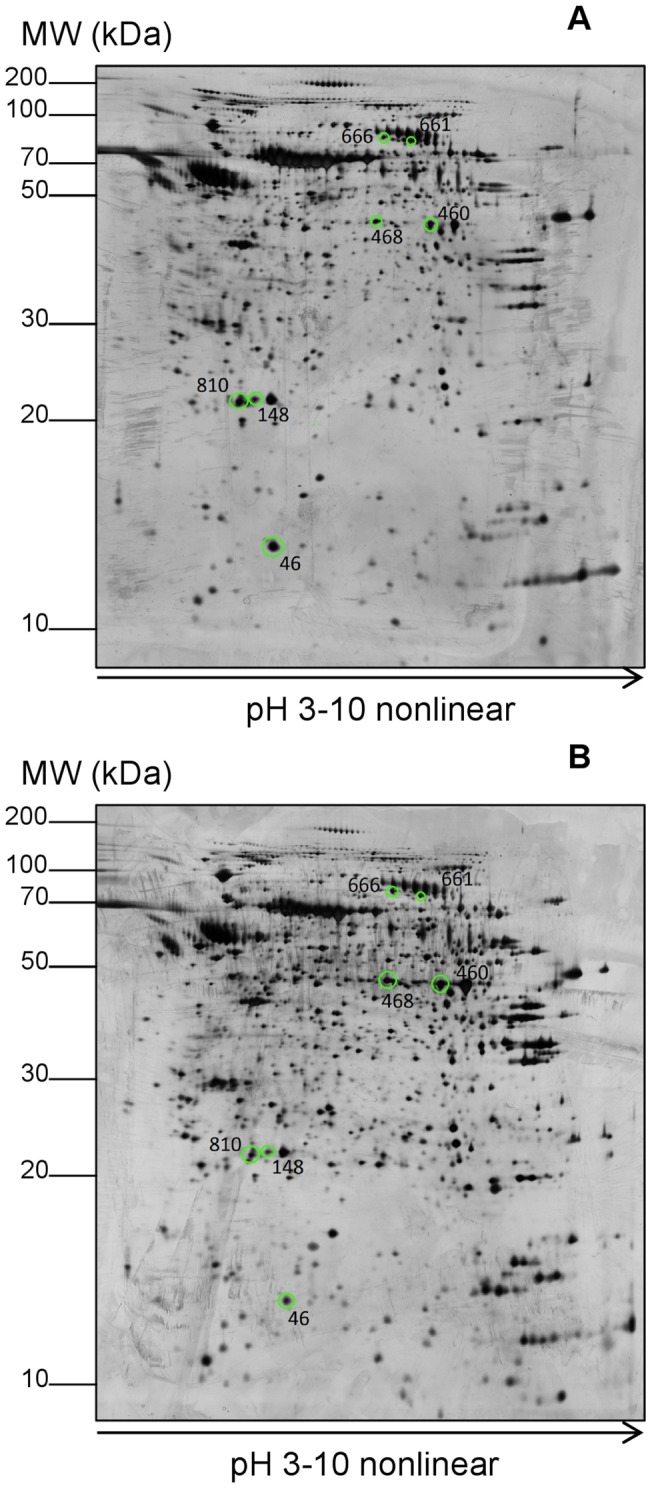
Analysis of proteins released from HepG2 cells. Two-dimensional gel electrophoresis (2D-GE) analysis of proteins released from HepG2 cells in control (A) and treatment with 60 µg/ml methanol extract of the *T. indica* fruit pulp (B). Analysis was performed on four independent biological replicates for proteins released from the control as well as treated cells. Approximately 1500 spots per gel within the pH 3–10 range were detected. Seven spots (circled and labeled) were differentially expressed (*p*<0.05) in which four were significantly up-regulated and three were significantly down-regulated.

**Table 1:** Mean percentage of spot volume of proteins that were differentially expressed.

Spot ID	Average Percentage of Volume ± SEM		*p*-value	Fold Change
	Control	Treated		
19*	0.223±0.058	0.074±0.015	0.0478	−3.0
46	0.731±0.205	0.289±0.136	0.0113	–2.5
148	0.214±0.014	0.096±0.014	<0.001	–2.2
284*	0.056±0.012	0.024±0.005	0.0426	−2.3
460	0.292±0.103	0.613±0.194	0.0265	+2.1
468	0.175±0.023	0.349±0.098	0.0136	+2.0
661	0.034±0.014	0.070±0.012	0.0084	+2.1
666	0.045±0.021	0.113±0.014	0.0018	+2.5
810	0.475±0.095	0.278±0.088	0.0227	–1.7

* Spots 19 and 284 were rejected as false positives after subjecting to FDR analysis using the Benjamini-Hochberg's method [10]. An adjusted p-value of less than 0.03125 was considered as statistically significant.

### Cell culture

The human hepatoma HepG2 cell line (ATCC, Manassas, VA, USA) was grown in a complete medium consisting of Dulbecco’s modified Eagle’s medium (DMEM) supplemented with 5 mM glucose, 10% foetal bovine serum (FBS; HyClone, Australia), 0.37% (w/v) sodium bicarbonate and 0.48% (w/v) HEPES, pH 7.4, in a CO_2_ humid incubation chamber at 37°C.

### Treatment of HepG2 cells with *T. indica* fruit pulp methanol extract

HepG2 cells were seeded at a density of 9.0×10^6^ in a 75 cm^2^ flask for 18–24 h followed by extensive washing with phosphate-buffered saline (PBS). The cells were then incubated for 24 h in serum-free medium in the presence of 0.02% DMSO (vehicle) as controls or a final concentration of 60 µg/ml methanol extract of *T. indica* fruit pulp. Secreted proteins were harvested from the cell culture media after 24 h incubation.

**Table 2:** Identification of differentially expressed proteins by MALDI-MS/MS.

Spot no	Protein description	SWISS-PROT Accession No.	MASCOT score	pI/MWϕ(kDa)	Av % of Vol ratio^a^	% Cov^b^	Matched Peptide Sequences
**46**	Transthyretin (TTR) precursor	P02766	252	5.52/15.88	−2.5	18	42–54; 55–68; 56–68
**148**	Apolipoprotein A-I (ApoA-I) precursor	P02647	216	5.56/30.76	−2.2	35	121–130; 132–140; 185–195
**460**	Alpha enolase (ENO1)	P06733	257	7.01/47.14	+2.1	16	16–28; 33–50; 184–193; 240–253; 270–281; 407–412
**468**	Rab GDP dissociation inhibitor beta (GDI-2)	P50395	618	6.11/50.63	+2.0	47	36–54; 56–68; 69–79; 90–98; 143–156; 194–208; 211–218; 279–288; 300–309; 310–328; 391–402; 403–418; 424–436
**661^c^**	Lactotransferrin precursor	P02788	46	8.50/78.13	+2.1	7	544–552
**666^c^**	Glutathione transferase omega-1	P78417	21	6.23/27.55	+2.5	8	12–25; 31–37
**810**	Apolipoprotein A-I (ApoA-I) precursor	P02647	437	5.56/30.76	−1.7	33	52–64; 121–130; 132–140; 165–173; 185–195; 231–239; 251–262

a Positive value signifies up-regulation against control samples and negative value signifies down-regulation in terms of fold-differences.

b % Coverage of the identified sequence.

c Considered not positively identified because of low MASCOT score (<55).

### Recovery of secreted proteins from cell culture media

Cell culture media containing the secreted proteins were centrifuged at 1000×*g* for 5 min and filtered through a 0.22 µm syringe filter to remove cellular debris. The supernatant was then concentrated using a Vivaspin column with a 5 kDa molecular weight cut off membrane (Sartorius Stedim, Germany) and centrifuged at 7000×*g* for 2 h. The concentration of protein was then determined using the Bradford assay kit (Bio-rad, Hercules, CA, USA).

### Determination of cell viability

Viability of cells cultured in serum-free media in the presence of the methanol extract of *T. indica* fruit pulp was estimated using 3-(4,5-dimethyl-2-thiazolyl)-2,5-diphenyl-2H-tetrazolium bromide (MTT) assay. Cells were plated at a density of 1.5×10^4^ cells per well in a 6-well plate and cultured in complete DMEM for 24 h. The cells were then washed with PBS three times and cultured in either complete or serum-free media with or without the methanol extract of *T. indica* fruit pulp. After 24 h, 100 μl of 5 mg/ml MTT (Merck, Germany) was added to each well. Cells were further incubated for 4 h and the MTT solution was then discarded. The precipitate in each well was then resuspended in 2 ml of isopropanol. The optical density (OD) of the samples was read at 570 nm.

**Figure 3:**
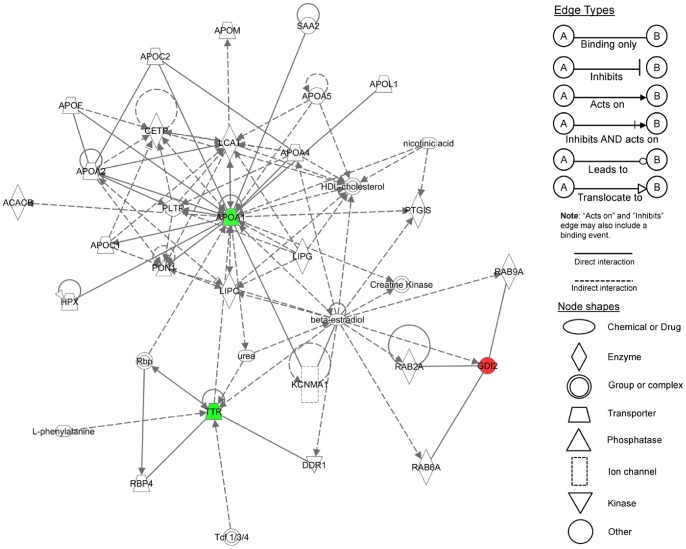
IPA graphical representation of the molecular relationships between differentially expressed proteins in HepG2 cells treated with *T. indica* extract. The network is displayed graphically as nodes (proteins) and edges (the biological relationships between the nodes). Nodes in red indicate up-regulated proteins while those in green represent down-regulated proteins. Nodes without colors indicate unaltered expression. Various shapes of the nodes represent the functional class of the proteins. The different arrow shapes represent different types of interactions. Edges are displayed with various labels that describe the nature of the relationship between the nodes. Names of proteins corresponding to the abbreviations are as follows: APOA1, Apolipoprotein A-1; APOA2, Apolipoprotein A-2; APOA4, Apolipoprotein A-4; APOA5, Apolipoprotein A-5; APOC1, Apolipoprotein C-1; APOC2, Apolipoprotein C-2; APOE, Apolipoprotein E; APOM, Apolipoprotein M; APOL1, Apolipoprotein L-1; ACACB, acetyl-CoA-carboxylase 2; SAA2, serum amyloid A2; LCAT, lecithin cholesterol acyltransferase; PLTP, phospholipid transfer protein; CETP, cholesterylester transfer protein; PTGIS, prostaglandin I synthase; RAB9A, Ras-related protein Rab 9A; RAB6A, Ras-related protein Rab 6A; GDI2, Rab GDP dissociation inhibitor beta; RAB2A, Ras-related protein Rab 2A; KCNMA1, Potassium large conductance calcium-activated channel, subfamily M, alpha member 1; DDR1, discoidin domain receptor tyrosine kinase 1; TTR, transthyretin; RBP4, retinol binding protein 4; LIPC, hepatic triglyceride lipase; LIPG, endothelial lipase; PON1, paraoxonase 1; HPX, haemopexin; Tcf 1/2/3, T-cell factor -1, -2, -3; Rbp, retinol binding proteins.

**Figure 4:**
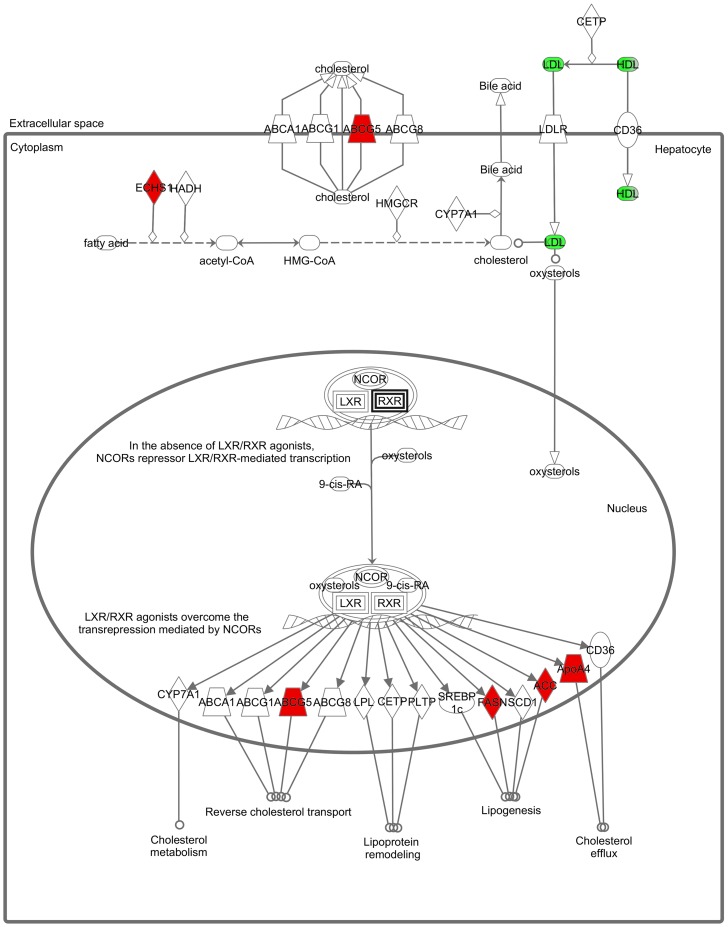
Predicted canonical pathway affected by *T. indica* fruit extract. IPA identified ‘LXR/RXR activation’ as the canonical pathway with the highest predicted potential/significance of being affected by the altered levels of TTR, ApoA-I and GDI-2 in HepG2 cells that were treated with *T. indica* fruit extract. Lines between the proteins represent known interactions. Red nodes indicate overexpression of genes induced by the extract, which was based on our previous report [8]. Abbreviation: ABCA1, ATP-binding cassette sub-family A member 1; ABCG1, ATP-binding cassette sub-family G member 1; ABCG5, ATP-binding cassette sub-family G member 5; ABCG8, ATP-binding cassette sub-family G member 8; ECHS, enoyl CoA hydratase; HADH, hydroxyacyl-CoA dehydrogenase; HMGCR, HMG-CoA reductase; CYP7A1, cytochrome P450, family 7, subfamily A, polypeptide 1; LDLR, low density lipoprotein receptor; CETP, cholesterylester transfer protein; CD36, cluster of differentiation 36; NCOR, nuclear receptor corepressor; LXR, liver X receptor; RXR, retinoid X receptor; 9-cis-RA, 9-cis-retinoic acid; LPL, lipoprotein lipase; PLTP, phospholipid transfer protein; SREBP-1c, sterol regulatory element-binding protein 1c; FASN, fatty acid synthase; SCD1, stearoyl-conzyme A desaturase 1; ACC, acetyl-CoA carboxylase; APOA4, apolipoprotein A-4; LDL, low density lipoprotein; HDL, high density lipoprotein.

### Two-dimensional gel electrophoresis (2D-GE)

Secreted proteins (40 µg) were first cleaned using the 2D clean-up kit (GE Healthcare, Piscataway, USA). The resulting protein pellet was then reconstituted in a rehydration solution, which contains 7 M urea, 2 M thiourea, 2% w/v CHAPS, 0.5% v/v IPG buffer, orange G, protease inhibitor. Immobiline pH gradient strips (13 cm, non-linear, pH 3–10, GE Healthcare, Uppsala, Sweden) were rehydrated for 18 h at room temperature in the presence of treated and untreated samples containing the same amount of proteins. Isoelectric focusing was performed under the following conditions: (i) 500 V, 1hr 10 mins, step and hold; (ii) 1000 V, 1 hr, gradient; (iii) 8000 V, 2 hrs 30 mins, gradient and (iv) 8000 V, 55 mins, step and hold. The temperature was maintained at 20°C and the current was kept at 50 µA per strip. Upon completion, strips were equilibrated in buffer containing 6M urea, 75 mM Tris-HCl, pH 8.8, 29.3% v/v glycerol, 2% w/v SDS, 0.002% w/v bromophenol blue and 1% w/v DTT for 15 min, followed by a second equilibration using the same buffer containing 4.5% w/v iodoacetamide instead of DTT for another 15 min. The second dimension separation was carried out at 15°C on 12.5% SDS slab gels using the SE 600 Ruby electrophoresis system (GE Healthcare, Uppsala, Sweden). The IPG strips were sealed on the top of the gels with 0.5% (w/v) agarose in SDS-electrophoresis buffer (25 mM Tris base, 192 mM glycine, 0.1% w/v SDS and a trace amount of bromophenol blue). SDS-PAGE was run at a constant power of 1 W/gel for 20 min, and then switched to 20 W/gel until the bromophenol blue marker was 1 mm away from the bottom of the gel. The gels were silver-stained with PlusOne Silver Staining Kit (GE Healthcare, Uppsala, Sweden) and scanned with the ImageScanner III (GE Healthcare, Uppsala, Sweden).

### Image and data analysis

Gel images were analysed using the ImageMaster 2D Platinum V 7.0 software (GE Healthcare, Uppsala, Sweden). Briefly, the 2D gel images were subjected to spot detection and quantification in the differential in-gel analyses module. To minimize variations between gels within the same group, protein spots were normalized using percentage of volume. Statistically significance (*p*<0.05, Student’s *t*-test) and presence in all 4 gels were the two criteria for acceptance of the differentially-expressed protein spots. Selected spots were filtered based on an average expression level change of at least 1.5-fold. The spots were then further subjected to false discovery rate analysis using Benjamini-Hochberg’s method to exclude false positive results [9].

### In-gel tryptic digestion

Differentially expressed protein spots were excised manually from 2-DE gels, and washed with 100 mM NH_4_HCO_3_ for 15 min. The gel plugs were then destained twice with 15 mM potassium ferricyanide/50 mM sodium thiosulphate with shaking. They were then reduced with 10 mM DTT at 60°C for 30 min and alkylated with 55 mM iodoacetamide in the dark at room temperature for 20 min. The plugs were later washed thrice with 500 µl of 50% ACN/ 50 mM NH_4_HCO_3_ for 20 min, dehydrated with 100% ACN for 15 min and dried using the SpeedVac. The gel plugs were finally digested in 6 ng/µl trypsin (Pierce, Rockford, IL USA), in 50 mM NH_4_HCO_3_ at 37°C for at least 16 h. Peptide mixtures were then extracted twice with 50% ACN and 100% ACN, respectively, and finally concentrated using the Speedvac until completely dry. The dried peptides were then kept at –20°C or reconstituted with 10 µL of 0.1% TFA prior to desalting using the Zip Tip C18 micropipette tips (Millipore, Billerica, MA, USA).

### Mass spectrometry and database search

Peptide mixtures were analysed by MALDI-TOF/TOF using an Applied Biosystems 4800 Plus MALDI-TOF/TOF (Foster City, CA, USA), after the trypsin digest were crystallized with alpha-cyano-4-hydroxycinnamic acid matrix solution (10 mg/ml, 70% ACN in 0.1% (v/v) TFA aqueous solution) and spotted onto a MALDI target (192-well) plate. The MS results were automatically acquired with a trypsin autodigest exclusion list and the 20 most intense ions selected for MS/MS analysis. Interpretation was carried out using the GPS Explorer software (Applied Biosystems, CA, USA) and database search using the in-house MASCOT program (Matrix Science, London, UK). Both combined MS and MS/MS searches were conducted with the following settings: Swiss-Prot database, *Homo sapiens*, peptide tolerance at 200 ppm, MS/MS tolerance at 0.4 Da, carbamidomethylation of cysteine (variable modification) and methionine oxidation (variable modifications). A protein is considered identified when a MASCOT score of higher than 55 and *p*<0.05 were obtained from the MS analysis.

### Functional Analysis by IPA

The proteomics data was further analysed using the Ingenuity Pathways Analysis (IPA) software (Ingenuity® Systems, www.ingenuity.com) to predict networks that are affected by the differentially expressed proteins. Details of proteins identified to be differentially released, their quantitative expression values (fold change difference of at least 1.5) and *p*-values (*p*<0.05) were imported into the IPA software. Each protein identifier was mapped to its corresponding protein object and was overlaid onto a global molecular network developed from information contained in the Ingenuity Knowledge Base. Network of proteins were then algorithmically generated based on their connectivity. Right-tailed Fischer’s exact test was used to calculate a *p*-value indicating the probability that each biological function assigned to the network is due to chance alone.

## Results

### Viability of HepG2 cells

To determine the influence of serum-free medium and the methanol extract of *T. indica* fruit pulp on viability of HepG2 cells, the MTT analysis was carried out. Our results showed no significant difference in viability of cells that were grown in complete or serum-free media (Figure 1A). Viability of HepG2 cells that were grown in the presence of the methanol extract of the *T. indica* fruit pulp at a final concentration of 60 µg/ml was also not significantly different from those grown in the control serum-free medium in the presence of 0.02% (v/v) DMSO (Figure 1B).

### 2D-GE analysis of proteins released from HepG2 cells

When culture supernatants of HepG2 cells were subjected to 2D-GE, more than 1500 highly resolved protein spots were detected in each gel. Figure 2 demonstrates typical 2D-GE resolved patterns of the proteins released from control (panel A) and *T. indica*-treated HepG2 cells (panel B). The percentage of volume contribution of each spot was then determined using the ImageMaster 2D Platinum V 7.0 software, and the fold change, if any, was acquired. Protein spots that were present in all gels (n = 4) and showed significant difference in their expression (p<0.05) in the culture media of treated and non-treated cells by more than 1.5 fold were initially selected. Based on these criteria, nine protein spots were initially found to be differentially expressed, four of which were significantly up-regulated and five significantly down-regulated. When the data were further subjected to false discovery rate (FDR) analysis based on the Benjamini-Hochberg’s method [9], two of the protein spots were categorized as false positives and excluded (Table 1). The seven proteins spots that were concluded to be differentially expressed when HepG2 cells were treated with *T. indica* were those designated with numbers 46, 148, 460, 468, 661, 666 and 810 as shown in Figure 2.

### Identification of differentially expressed proteins

Among the seven protein spots that were altered in expression, five spots (46, 148, 460, 468 and 810) were identified by mass spectrometry and database search. Spots 661 and 666 were not successfully identified by MS/MS analysis as their scores were lower than the cut off value for positive inclusion criteria. This could probably be due to their close proximity to the high abundant proteins which hinder the detection of their peptides. The five differentially expressed proteins that were identified include transthyretin (TTR – spot 46), two isoforms of apolipoprotein A-I (ApoA-I – spots 148 and 810), alpha enolase (ENO1 – spot 460) and rab GDP dissociation inhibitor beta (GDI-2 – spot 468) (Table 2). ENO1 and GDI-2 were apparently up-regulated by approximately 2-fold, while TTR was down-regulated by 2.5-fold. The two spots identified as ApoA-1 was down-regulated by 2.2- and 1.7-folds.

### Pathway interactions and biological process analysis

Analysis using IPA identified “Lipid Metabolism, Molecular Transport and Small Molecule Biochemistry" as the sole putative network linking three of the differentially expressed proteins with other interactomes, with a score of 9. A score of 2 or higher indicates at least a 99% confidence of not being generated by random chance and higher scores indicate a greater confidence. Figure 3 shows a graphical representation of the predicted molecular relationships between ApoA-I, TTR and GDI-2. The network suggests *T.indica* fruit pulp extracts affect lipid metabolism, converging on beta-estradiol. A canonical pathway analysis ranked the LXR/RXR activation with the highest significance (*p*<1.29×10^−04^; Figure 4).

## Discussion

In this study, proteomic techniques were used to analyze the expression of proteins that were released by HepG2 cells in response to treatment with the methanol extract of *T. indica* fruit pulp. Serum-free DMEM culture media from HepG2 cells grown for 24 hours in the absence and presence of *T. indica* fruit pulp extract were initially subjected to 2D-GE. The use of serum-free medium was necessary for the proteomic analysis to avoid masking of the proteins released by the cells by those present in FBS. The results of our MTT assays showed that viability of the HepG2 cells was not affected by use of serum-free medium and neither was it significantly different when the cells were exposed to the *T. indica* fruit pulp extract.

Among the thousands of protein spots that were detected in the 2D-GE profiles of culture media isolated from HepG2 cells grown in the absence or presence of the methanol extract of *T. indica* fruit pulp, only seven were found to be altered in expression. Five of the protein spots were found to be those of TTR, ENO1, GDI-2 and ApoA-I (2 isoforms), whilst two spots were not successfully identified Exposure of the HepG2 cells to the *T. indica* fruit pulp extract appeared to have caused the increased release of ENO1 and GDI-2 but decreased secretion of TTR and ApoA-I. While the two latter proteins are known to be secretory proteins, ENO1 and GDI-2 are apparently cytosolic proteins [10]. However, several earlier studies had also detected the presence of ENO1 and GDI-2 in the culture media of HepG2 cells [11], [12].

When the differentially expressed proteins were subjected to analysis using IPA, all but ENO1 were found to be interconnected with interactomes in lipid metabolism. ENO1, although more popularly known as a glycolytic enzyme, is apparently a multifunctional protein that also acts as a receptor, activator and regulator molecule [13], [14]. Hence, the ENO1 that was detected in the culture media in this study may not be involved in glycolysis. Due to the multiple roles played by ENO1, it is difficult to speculate the reason why the release of the protein was increased when HepG2 cells were exposed to the *T. indica* fruit pulp extract.

Interestingly, the three differentially expressed proteins that are involved in lipid metabolism appeared to be commonly associated with the same hormonal regulation, i.e., estradiol, and the homeostasis of cholesterol. GDI-2 functions to translocate prenylated Rab proteins from the cytosol to the membrane to form nascent transport vesicles. The protein also assists the subsequent retrieval of Rab proteins [10], [15], which are key regulators for the transport of lipids and proteins between cell organelles from target membranes [15], [16], [17]. To date, approximately 70 Rab proteins had been identified but their specific functions are still largely unknown [18]. These include Rab11, whose overexpression has been shown to block the recycling of cholesterol from the endosome recycling compartment to the plasma membrane [19], [20]. On the other hand, Rab8 has been shown to assist the redistribution of cholesterol from late endosomes to the cell periphery and stimulate cholesterol efflux through the ABCA1/ApoA-I pathway [21]. The increased release of GDI-2 by HepG2 cells when they were exposed to the methanol extract of *T. indica* fruit pulp was probably to enable the recycling of Rab proteins that are involved in the cholesterol homeostasis in the cell.

TTR is a protein that is mainly synthesized in the liver and the choroid plexus of the brain [22]. Its two main functions are to transport thyroxine and retinol through binding to the hormone and retinol-binding protein, respectively [23], [24]. However, a small fraction of plasma TTR (1–3%) is apparently associated with ApoA-I, the major apoprotein found in the anti-atherogenic lipoprotein HDL. ApoA-I is synthesized in the liver (as well as the intestine) and its secretion is believed to be either in a lipid-free/poor or pre-lipidated forms (intracellularly assembled nascent HDL) [25]. Ohnsorg [26], Liz [27] and their coworkers showed that TTR cleaves the C-terminus of ApoA-I, which is necessary for the transport of lipid-free ApoA-I through the aortic endothelial cells. When HepG2 cells were exposed to the tamarind extract in this study, secretion of both TTR and ApoA-I was reduced by more than 2-fold. The two proteins were also shown to be interconnected via our IPA analysis. This alteration reflects the indirect effects of *T. indica* fruit pulp extract in regulating the function of HDL in the reverse transport of cholesterol and also in line with the earlier findings on the cholesterol and triacylglycerol lowering effects of the fruit extract in hypercholesterolaemic hamsters [4] and in humans [5].

In our recent report, we have also described the up-regulated expression of the *APOA1* gene by 1.2 fold when HepG2 cells were exposed to the same concentration of *T. indica* fruit pulp extract [8]. The high ApoA-I mRNA levels that were detected in HepG2 cells upon exposure to the extract as opposed to the low levels of ApoA-I that appeared in the media could be either due to the decrease in the rate of export of the apoprotein or that the mRNA may not be translated into ApoA-I. Our previous study also showed that the *T. indica* fruit pulp extract induced the expression of the *ABCG5* gene. ABCG5 apparently forms a dimer with ABCG8 to assist the secretion of cholesterol into the bile and its subsequent excretion via the faeces. Therefore, the lower ApoA-I secretion that was detected in the present study suggests that the *T. indica* fruit pulp extract may be promoting the excretion of cholesterol via the ABCG5 instead of the ApoA-I transport system. Interestingly, the ABCG5/8-mediated cholesterol excretion and absorption and ABCA-1-mediated cholesterol efflux are apparently controlled by the liver X receptors, LXRs. Earlier studies have apparently shown that proanthocyanidins, which constitutes more than 73% of the total phenolic content of *T. indica* extract [6], were able to modulate the activation of LXR/RXR [28]. Hence, the data of this study, when taken together with that of our earlier report, suggest that the *T. indica* fruit pulp extract exerts its lipid lowering effects through the modulation of the LXRs, a conception that was similarly derived from the canonical pathway analysis.

### Conclusion

The methanol extract of *T. indica* fruit pulp altered the release of ENO1, ApoA-I, TTR and GDI-2 from HepG2 cells. Our results provide support on the predicted effect of *T. indica* extract on cellular lipid metabolism, particularly that of cholesterol.
